# Indoor Volatile Organic Compounds: Concentration Characteristics and Health Risk Analysis on a University Campus

**DOI:** 10.3390/ijerph20105829

**Published:** 2023-05-16

**Authors:** Shengjia Jin, Lu Zhong, Xueyi Zhang, Xinhe Li, Bowei Li, Xuekun Fang

**Affiliations:** 1 College of Environmental & Resource Sciences, Zhejiang University, Hangzhou 310058, China; 2 Center for Global Change Science, Massachusetts Institute of Technology, Cambridge, MA 02139, USA

**Keywords:** indoor VOCs, university campus, concentration characteristics, health risks, seasonal variation

## Abstract

Volatile organic compounds (VOCs) are major indoor air pollutants that contain several toxic substances. However, there are few studies on health risk assessments of indoor VOCs in China. This study aimed to determine the concentration characteristics of VOCs on college campuses by collecting VOC samples from different locations on campus during different seasons combined with the exposure times of college students in each location obtained from a questionnaire survey to assess the possible health risks. The highest total VOC concentration (254 ± 101 µg/m^3^) was in the dormitory. The seasonal variation of TVOC concentrations was related to the variation of emission sources in addition to temperature. Health risk assessments of VOCs were evaluated using non-carcinogenic and carcinogenic risk values, represented by hazard quotient (*HQ*) and lifetime cancer risk (*LCR*), respectively. The non-carcinogenic risks at all sampling sites were within the safe range (*HQ* < 1). Dormitories had the highest carcinogenic risk, whereas the carcinogenic risk in the other three places was low (with *LCR* < 1.0 × 10^−6^). Moreover, 1,2-dichloroethane was identified as a possible carcinogenic risk substance in the dormitory due to its high *LCR* (1.95 × 10^−6^). This study provides basic data on health risks in different locations on campus and a basis for formulating measures to improve people’s living environments.

## 1. Introduction

People spend 80–90% of their time indoors every day [1,2,3], and indoor air quality (IAQ) has a significant impact on human health. Indoor air pollution is estimated to cost more than two million disability-adjusted life years (DALY) annually in Europe [4]. In addition, owing to the increase in energy-saving requirements for buildings, the airtightness of buildings inhabited by humans has increased [5]. Therefore, the reduction in air circulation may increase the concentration of pollutants and lead to increased health risks in indoor environments.

Volatile organic compounds (VOCs) are organic gases with a boiling point between 50 and 260 °C [6] and are important indoor air pollutants. Exposure to VOCs poses a considerable health concern owing to its potential associated chronic and acute health risks [7], resulting in symptoms similar to those of sick-building syndrome (SBS), including sensory and lung irritation, chronic obstructive pulmonary disease (COPD), asthma, and cancer [2,7,8,9,10,11]. Indoor sources of VOCs include personal care products [12], cooking [13], cleaning products [14], the respiratory metabolism of occupants [15,16], and volatile gases from building materials [17]. The emission of VOCs from these sources depends on a variety of factors [18]. For instance, higher temperatures increase the initial acetaldehyde emissions and accelerate the decrease in later emissions, whereas higher humidity results in greater values of the emission factor [19].

University campuses are the main places where young people study and live. Owing to the development of education in China, the number of university enrollments is increasing [20]. However, studies on VOCs in universities are limited. Kumar et al. [21] have conducted a health risk assessment of indoor VOCs in the university library of Delhi, India, and found that the cancer risk associated with benzene (8.93 × 10^−6^) exceeded the value (1 × 10^−6^) recommended by the United States Environmental Protection Agency (USEPA) [21]. Jiang et al. [20] have measured VOCs in three universities and found that the tested buildings have more than a 90% probability of exceeding the cancer risk. Kang et al. [1] collected samples from the dormitory, teaching building, and library of a university, and found that the total concentration of VOCs in the dormitory was higher than the Chinese indoor environmental standard (0.6 mg/m^3^). Additionally, there are more indoor pollution sources in universities [22]. Therefore, it is essential to focus on IAQ on the university campus.

In this study, samples were collected from the dormitories, classrooms, canteens, and libraries of the Zijingang Campus of Zhejiang University for four months. A total of 116 VOCs were quantified and combined with the survey results on the living habits of college students to assess the health risks of VOCs in different indoor environments on college campuses. This study provides insights into understanding the health risks of VOCs in the study and living environments of college students, providing a reference for improving the indoor environment.

## 2. Methods

### 2.1. Sample Collection

This study conducted sampling activities from April 2022 to January 2023 during four months (namely, April, July, October, and January). Samples were collected in the classroom, dormitory, canteen, and library for 3–4 consecutive days each month. The sample sizes for each site are detailed in Table S1. One classroom and the first floor of the library were chosen randomly, and samples were collected from each of them at 12:00 and 16:00 local time (LT) on each sampling day. The living place was chosen at random from a dormitory room in the dormitory park and the second floor of the East District Canteen; the dormitory collection times were 5:00 and 21:00 LT on each sampling day, and the canteen collection time was 18:00 LT. The sampling was done during the population’s most frequent exposure. The sampling point was fixed during the sampling campaign. The samples were collected with vacuum stainless steel canisters (3.2 L, Entech, Simi Valley, PA, USA) at 1.5 m above the ground (generally at the height of breathing) in the center of the room. Given the limited samples of canteens and libraries, the results of these two places in this study are for reference only.

### 2.2. Sample Analysis

The samples were analyzed using a gas chromatography–flame ionization/mass spectrometry detector (GC–FID/MSD) (ZF-PKU-VOC1007, Beijing Pengyu Changya Environmental Protection Technology Co., Ltd., Beijing, China). Detailed analytical procedures can be obtained from the previous study [23]. Offline samples were pumped at a flow rate of 10 mL/min for 30 min into a cryogenic trap at −160 °C (ZF-PKU-VOC1007). The analyte was then heated, released at 110 °C, and transferred with the carrier gas to a dual-channel gas chromatography system. The C_2_–C_5_ hydrocarbons were separated via DM-PLOT columns (15 m × 0.32 mm × 5 μm, Agilent Technologies, Santa Clara, CA, USA) and then detected using FID. The C_5_–C_12_ hydrocarbons, halogenated hydrocarbons, and oxygenated organic compounds (OVOCs) were separated using a DB-624 column (60 m × 0.25 mm × 1.4 μm, Agilent Technologies, Santa Clara, CA, USA) and detected using MSD. Finally, the analytical assay system used in this study quantified 116 VOCs, including 58 non-methane hydrocarbons (NMHCs) (alkanes, alkenes, alkynes, and aromatics), 35 halogenated hydrocarbons, 21 OVOCs, and 2 other compounds (i.e., carbon disulfide and acetonitrile).

### 2.3. Health Risk Calculation

Based on the VOC data measured in the experiment, this study evaluated the lifetime cancer risk (*LCR*) and hazard quotient (*HQ*). The *LCR* value indicates the probability of cancer occurrence and is usually expressed in terms of the number of individuals with cancer in a certain population. For example, the carcinogenic risk value is 10^−6^–10^−4^, that is, one cancer patient will be added for every 10,000 to 1 million people [24]; *HQ* is the value obtained by dividing the average exposure concentration by the reference concentration, indicating the maximum amount of pollutants that will not cause adverse reactions in the human body ingested per unit weight per unit time. The average daily dose of any compound (*E_D_*, mg·kg^−1^ day^−1^) was calculated using Equation (1) [25]. The effective annual exposure (*E_Y_*) in Equation (2) and the effective lifetime exposure (*E_L_*) in Equation (3) was used to calculate the chronic non-cancer *HQ* and *LCR*, respectively [26]:

(1) $$E_{D}=(CA\times IR\times ET)/BW$$

(2) $$E_{Y}=E_{D}\times EF/365$$

(3) $$E_{L}=E_{Y}\times ED/AT$$

where *CA* is the average concentration of air pollutants (mg·m^−^^3^), *IR* is the inhalation rate (0.83 m^3^·h^−^^1^ [25,26]), *ET* is the exposure time (hours day^−^^1^), *BW* is the weight (64.3 kg [27]), *EF* is the exposure frequency (225 days/year, based on the average time college students are in school), *ED* is the exposure duration (4 years, based on the average time college students are in college), and *AT* is the average life span (74.875 years [28]).

The non-carcinogenic risk of VOCs is expressed as *HQ*, which is defined as the ratio of the annual average daily dose (*E_Y_*) to the reference dose *RfD*. The *LCR* was evaluated by multiplying the effective lifetime exposure (*E_L_*) by the slope factor (*SF*):

(4) $$HQ=E_{Y}/RfD$$

(5) $$LCR=E_{L}\times SF$$

*RfD* (mg kg^−^^1^ day^−^^1^) is the level at which adverse health effects are unlikely to occur, and *SF* is the slope factor or carcinogen potency slope (mg^−1^ kg·day). All exposure parameters, *RfD* and *SF*, for each pollutant used in the analysis were obtained from the USEPA [25,29] (Tables S2 and S3).

Inhalation exposure during the year was used to assess the carcinogenic and non-carcinogenic risks of toxic VOCs in the teaching building, library, dormitory, and canteen, respectively. A non-carcinogenic risk value greater than 1 indicates that the concentration of toxic VOCs in the area poses a non-carcinogenic risk to residents, and a value less than 1 indicates no risk. However, some researchers have found that compounds with non-carcinogenic risks higher than 0.1 may also have potential non-carcinogenic risks [30]. For cancer risk, a compound with a risk value >1.0 × 10^−^^4^ is considered an “identified risk”, a compound with a risk value between 1.0 × 10^−^^5^ and 1.0 × 10^−^^6^ is a “possible risk”, and a compound with a risk value less than 1.0 × 10^−^^6^ is a negligible risk [24]. Species with potential risks were also presented in this study.

A total of 31 questionnaires were collected for this study. The results show that college students who were investigated spent 7.5 h a week in the canteen, 94.9 h in the dormitory, 40.5 h in the classroom, and 3.2 h in the library.

## 3. Results

### 3.1. VOCs Concentration Characteristics at Different Locations

**Figure 1:**
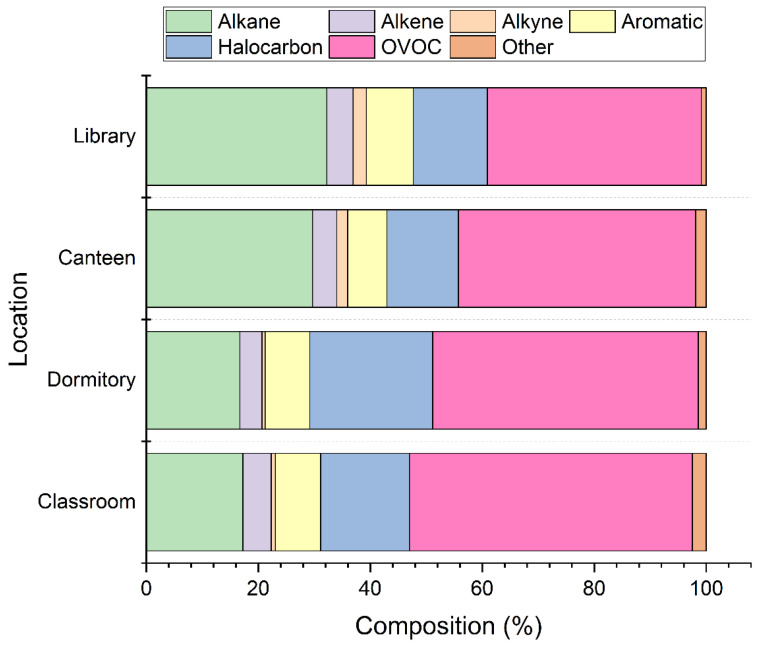
Composition of alkane, alkene, aromatic, alkyne, halocarbon, OVOC, and other species in the four sampling locations during the whole sampling period.

During the sampling period, the total concentration of VOCs (TVOCs) in the dormitory was the highest (254.3 ± 100.8 μg/m^3^), followed by the canteen (183.6 μg/m^3^), classroom (154.0 ± 67.5 μg/m^3^), and library (139.4 μg/m^3^). The proportions of the different types of VOCs in the four locations were similar (Figure 1). At the four sites, the main chemical composition was OVOC, accounting for 38–51%. Except for the proportion of halocarbon (22 ± 7.3%) in the dormitory secondary to OVOC, the abundance of alkanes (17–27%) was the second most abundant group among the other three sites. Volatile halocarbons can be emitted by chlorine disinfectants in tap water and detergents containing chlorides [31,32,33]; therefore, the higher levels of halocarbons in dormitories may be related to the increased use of disinfectants and detergents.

**Figure 2:**
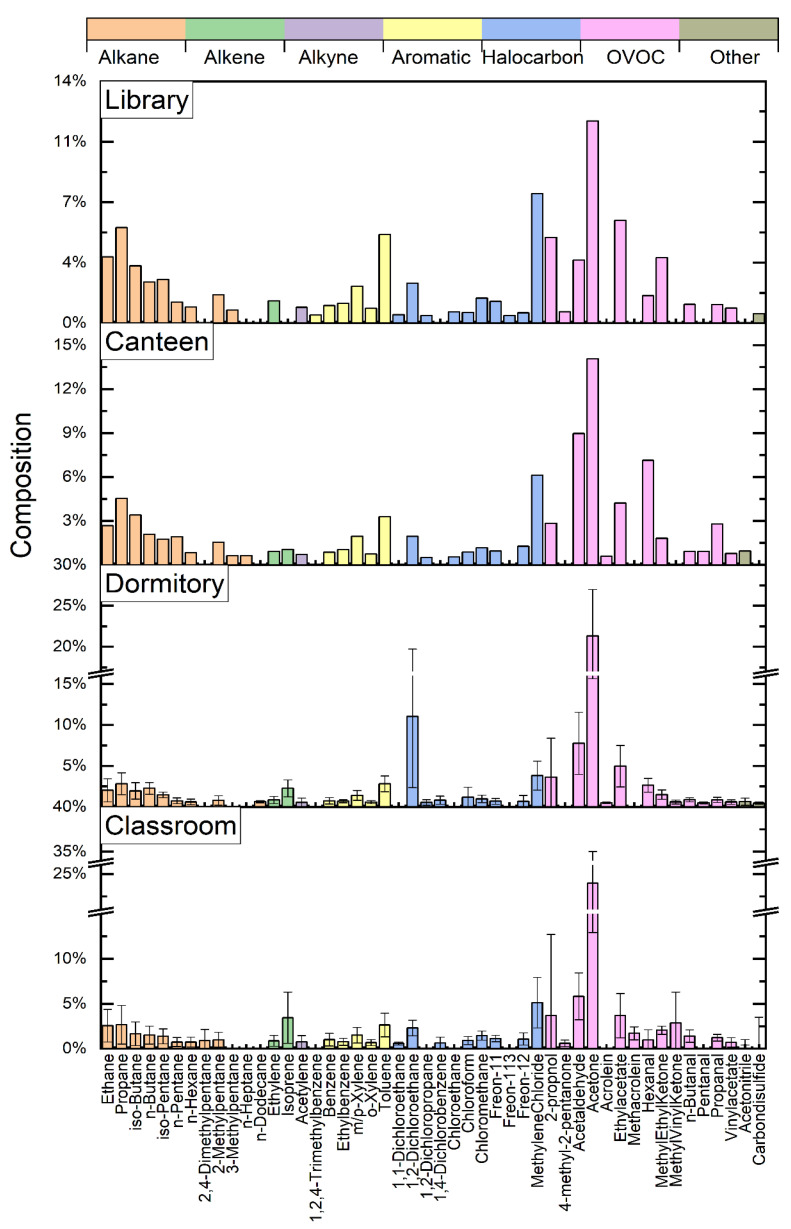
The average proportion of the top 39 VOC substances at different locations. Due to only 1 sample being collected in the canteen and the library, the results in both places have no error bars.

The average concentration level of each species during the whole sampling period at the four places is listed in Table S4. Figure 2 shows the average proportion of the top 39 VOCs from different locations, accounting for 88.5–89.7% of the total VOC concentration, among which acetone (12–24%) had the highest percentage. The health effects of acetone have been extensively studied; if inhaled, the compound is usually classified as having low acute and chronic toxicity. Moreover, exhaust fumes from the outer layer of furniture paint, organic solvents used daily (e.g., detergents), and personal care products can raise indoor acetone levels [34,35,36]. In addition, in a well-ventilated indoor environment, VOCs emitted by people (a major source of acetone [37]) account for approximately 57% of the total concentration of VOCs distributed in the indoor air [15]. Therefore, the abundant acetone in the four indoor environments may be related to consumer products and human metabolism.

In addition, we observed differences in the chemical profiles of the 39 VOCs across the four places, suggesting differences in emission sources. For example, the proportion of C_2_–C_5_ alkanes in the library and canteen were higher than that in the dormitory and classroom (Figure 2). C_2_–C_5_ alkanes are mainly generated by fuel combustion, among which ethane and propane are maker species in natural gases used for heating or cooking [38,39], and isopentane mainly originates from traffic-related sources [40], which can enter the room through natural ventilation, mechanical ventilation, and infiltration [41]. Given the proximity of the library to the main road and the consumption of natural gas for cooking in the canteen, the higher proportion of C_2_–C_5_ in the library and canteen was within expectations. Moreover, a higher abundance of 1,2-dichloroethane was identified in the dormitory compared to the other places (11 ± 8.7% compared to 1.9–2.4% at the other three places), suggesting stronger emissions of 1,2-dichloroethane in the dormitory [42,43,44,45], which are discussed in detail below in Section 3.3.

### 3.2. Temporal Variation of VOCs

In this study, we used samples collected in January, April, July, and October to characterize VOCs in winter, spring, summer, and autumn, respectively. However, due to the limited samples collected in the canteen and library, it was not possible to analyze the seasonal changes in these two locations.

**Figure 3:**
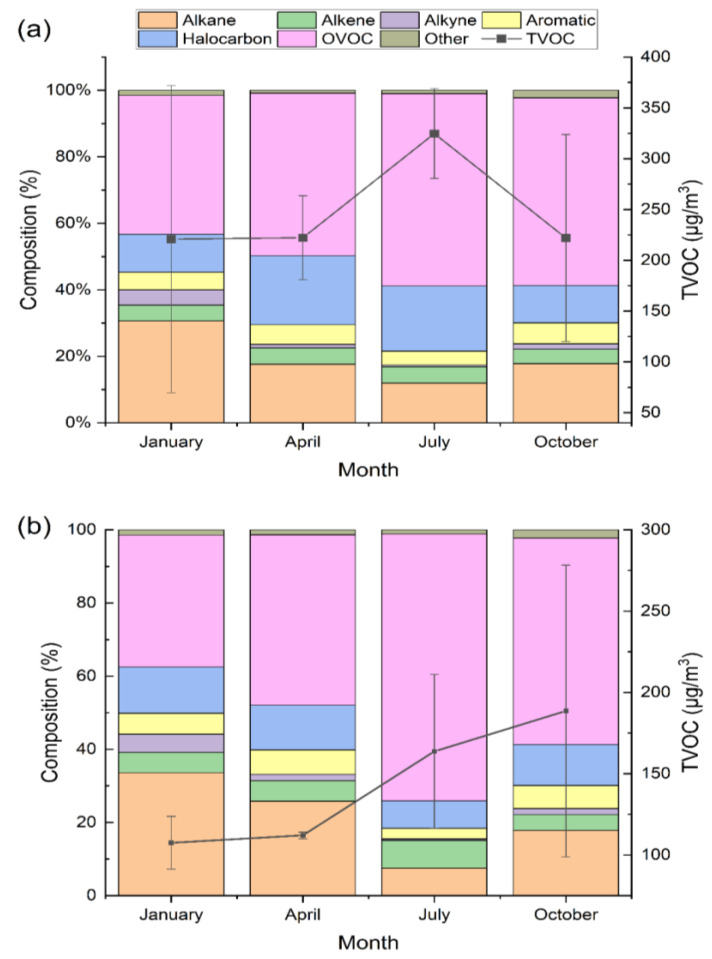
Composition of major chemical groups and average concentration of TVOCs in January, April, July, and October in the dormitory (**a**) and classroom (**b**), respectively.

The seasonal variation in the average concentration of TVOCs was not consistent in the classroom and dormitory (Figure 3). The average concentration of TVOCs in summer (324.7 ± 44.2 μg/m^3^) in the dormitory was higher than that in the other three seasons (220–223 μg/m^3^). In the classroom, the mean concentration of TVOCs was highest in autumn (188.6 ± 89.7 μg/m^3^), followed by summer (163.7 ± 47.4 μg/m^3^). According to the outdoor temperature measured by a weather station (30.14° N, 120.1° E) in Hangzhou, the indoor TVOC concentrations increased with outdoor temperature (Pearson’s r = 0.55, Figure S1), which is consistent with previous studies that higher temperature favors VOC volatilization [18,35]. Therefore, higher concentrations of TVOCs in summer and autumn (with temperatures of 26.8 ± 0.9 °C and 19.0 ± 5.0 °C, respectively) were in line with our expectations. However, the higher concentration of TVOCs in dormitories in summer may be attributed to the effect of high temperatures in summer and the combined effects of other factors (such as ventilation rate, humidity, human activities, etc.), which warrants further research. In addition, the concentration levels of TVOCs in the afternoon (205.5 ± 93.6 μg/m^3^) were comparable to those in the morning (200.0 ± 103.2 μg/m^3^), suggesting constant VOC emissions in indoor air during the day.

As for individual species, the average concentration of ethane and propane, which were mainly related to natural gas consumption [46], was higher in winter (7.7 ± 2.0 (6.1 ± 1.4) μg/m^3^ and 8.4 ± 3.3 (6.3 ± 2.0) μg/m^3^, respectively) than that in summer (2.8 ± 0.6 (1.4 ± 0.3) μg/m^3^ and 6.0 ± 1.5 (0.9 ± 0.2) μg/m^3^, respectively) in the dormitory (classroom), reflecting the variation in the strength of heating sources. Human breath and biogenic sources were sources of isoprene [47,48,49]; in summer, the average concentration of isoprene (9–12 μg/m^3^) was higher than in the other seasons (0.9–4.7 μg/m^3^). Considering human breath to be constant in general, the obvious high concentration of isoprene in the dormitory (9.3 ± 2.2 μg/m^3^) and classroom (11.6 ± 3.1 μg/m^3^) in summer indicates the obvious impact of biogenic sources (although the biological source is mainly outdoors, it enters the room with air circulation) on indoor air. Species mainly associated with solvent use or household products (such as m,p-xylene, toluene, octane, etc.) [50,51,52] did not show consistent seasonal variation in dormitories and classrooms, suggesting that indoor emissions of these substances are more likely to be randomly interred by anthropogenic activities.

For the composition of major chemical groups, the proportion of alkanes in winter (22.2 ± 4.5%) and autumn (22.3 ± 2.0%) in the dormitory were approximately two-fold higher than those in spring and summer. In contrast, high and low abundances of alkanes in the classroom were observed in winter (27.3 ± 4.5%) and summer (8.6 ± 1.2%), respectively. The abundant alkanes in winter in the classroom and dormitory may be related to the increased demand for natural gas for heating. Outdoors, the proportion of OVOCs usually increases in summer along with increases in stronger photochemical reactions [53]. In this study, the maximum proportion of OVOCs was observed in the summer both in the dormitory (49.3 ± 3.7%) and classroom (64.9 ± 2.1%), which suggests the impact of the outdoor environment on indoor VOCs. A higher proportion of OVOCs in the classrooms than in the dormitories in summer may be related to more crowded populations (indicating greater metabolic emissions) and better ventilation rates (indicating the greater influence of the outdoor environment on indoor air).

### 3.3. Health Risk Analysis

#### 3.3.1. Non-Carcinogenic Risk Analysis of VOCs

There were no substances with non-carcinogenic risks at any of the four sites during the sampling period, as the *HQ*s of the various substances were less than 1 (Table S2). The top 5 substances in terms of *HQ* in classrooms, dormitories, canteens, and libraries all contained acrolein, benzene, chloroform, and trichloroethylene (Table 1). The non-carcinogenic risk value for acrolein was the highest in each of the four sites and greater than 0.1 in the dormitory. Acrolein is a known respiratory toxicant and one of the 188 most dangerous air pollutants identified by the US Environmental Protection Agency (Washington, DC, USA). Studies have shown that outdoor air contributes little to acrolein and that wood and cooking are potential sources of acrolein indoors [54,55,56]. Therefore, wood-based furniture and desks in the dormitory, classroom, and library and the cooking activity in the canteen are the possible sources of acrolein in this study.

#### 3.3.2. Carcinogenic Risk Analysis of VOCs

Based on the average concentration of each toxic VOC, the *LCR* value of each toxic substance during the sampling period was calculated (Table S3). In this study, most of the top 5 compounds according to the *LCR* were halocarbons, indicating that halocarbons have a non-negligible impact on human health in indoor air (Table 1). The compound 1,2-dichloroethane had the highest *LCR* in all the sampling sites, which was also found in the indoor environment of an industrial area in Taiwan in a previous study [57]. There was no carcinogenic risk in the classroom, canteen, and library during the sampling period, as the *LCR* values for each carcinogen were less than 1 × 10^−6^, indicating that 1,2-dichloroethane poses a possible carcinogenic risk to students living in this environment for four years. However, the average *LCR* values of 1,2-dichloroethane in dormitories (1.96 × 10^−6^) exceeded the safety threshold (1 × 10^−6^). Human exposure to 1,2-dichloroethane is primarily through inhalation in urban or industrial areas [57,58]. Owing to the lack of obvious industrial sources around the study area and evidence that indoor sources of 1,2-dichloroethane are increasing [59], we speculate that 1,2-dichloroethane in this study mainly originates from indoor sources. During the sampling period in this study, solvents such as laundry detergent and bleach were placed in the dormitory, and toilet cleaners, deodorants, and other reagents were used for cleaning. Moreover, the residences and bathrooms were not separated, and the ventilation of the bathroom was inadequate, thereby resulting in a high health risk related to 1,2-dichloroethane in the dormitory. In addition, the maximum tolerated concentration of each toxic substance was calculated for students living on campus for 4 years based on the identified carcinogenic risk of 1 × 10^−4^ (Table S3), and the maximum tolerated concentration of each toxicant was higher than that of its corresponding average measured concentration by 1–5 magnitudes. Therefore, the campus in this study did not pose a threat to human health, but it should be noted that the results are only for people who have been exposed to it for 4 years, and the results for people with other exposure times will vary.

## 4. Discussion

This study provides an overview of the seasonal variation in the fractions and concentrations of indoor VOCs in different locations on campus (dormitory, classroom, library, and canteen) and an assessment of their associated health risks. Although there are previous studies on this topic, they are few in number and incomplete. Some studies have focused on VOC pollution within study sites (e.g., libraries, laboratories, and classrooms) and neglected the living places of students (e.g., dormitories and canteens) [44,60,61,62]; some studies have focused on a few specific VOC substances (e.g., carbonyl compounds, BTEX, formaldehyde, etc.) [36,61] or detected only a few dozen VOC substances [9,22,62]. In the present study, we covered the living places that college students are often exposed to as well as study places and detected a total of 116 VOCs, which is more comprehensive than previous studies.

Compared with other measurements conducted in universities, the average indoor TVOC concentration (203.3 ± 97.7 μg/m^3^) in this study was considerably lower than those measured by Kang et al. [1] in Tianjin, China (120–1620 μg/m^3^), by Akal et al. [9] in Ankara (770–2650 μg/m^3^), and by Mundackal and Ngole-Jeme [2] in South Africa (260–1062 μg/m^3^). The lower TVOC concentrations observed in our study may be attributed to several causes. First, they may be related to the environment around the sampling location; Akal et al. [9] conducted sampling in laboratories, where a large amount of volatile chemical reagents is usually consumed or stored. However, in our study, sampling was mainly conducted in the classroom and dormitory, where VOC emissions are usually lower than those in laboratories. Second, they may also be related to the age or renovation activities of the buildings to be measured; Kang et al. [1] collected samples at a university that had just been renovated two months ago, whereas the buildings investigated in the present study had been renovated more than two years ago. Finally, differences in the number of compounds quantified in different studies will also lead to differences in TVOC concentrations. For example, as many as 568 compounds were detected by Kang et al. [1], but only 116 compounds were detected in this study.

In the present study, we also focused on assessing the health risk associated with VOCs. Benzene series, especially benzene, toluene, ethylbenzene, and xylene (commonly known as BTEX) have received extensive attention in the health risk assessment of indoor VOCs [20,63]. In our study, the average concentrations of benzene, toluene, ethylbenzene, and xylene were 1.53 ± 0.84 μg/m^3^, 5.76 ± 3.89 μg/m^3^, 1.46 ± 0.81 μg/m^3^, and 4.18 ± 2.57 μg/m^3^, respectively, and that of BTEX was higher in the dormitory than in the classroom. These observed concentrations were lower compared to those reported in other studies conducted on university campuses [9,20,21,56,64,65,66] (Table 2).

Specifically, the concentration of benzene was considerably lower on our campus than the average level of the three campuses in China that had just been renovated within two years (benzene was as high as 97.5 μg/m^3^) [20]; however, it was comparable to that in a newly renovated office in a campus in Poland (1.13 ± 0.66 µg/m^3^) [66], which may be related to the varied benzene content of the building materials used in different campuses. Moreover, compared to indoor places other than university campuses, the concentration levels of benzene in this study were comparable to those reported in many primary and secondary schools (0.11–5.93 µg/m^3^ in Bari, Italy [68]; 0.94 ± 1.6 µg/m^3^ in Ho Chi Minh, Vietnam [69]; and 1.67 ± 1.25 µg/m^3^ in Gliwice, Poland [71]), slightly higher than the average concentration in the residential homes in Ashford, UK (0.5 (0.2–1.8) µg/m^3^) [74] and Louisiana, USA (1.14 (0.04–13.57) µg/m^3^) [75], and lower than those in electromechanical repair and car painting centers (59.2 µg/m^3^) [73] and residential homes near an industrial park (7.0 ± 4.1 µg/m^3^) [76]. The difference in the concentration of benzene in these different types of places may be related to the surrounding environment and the intensity of indoor emission sources. Moreover, the discrepancies for toluene, ethylbenzene, and xylene between this study and other studies were similar to those of benzene (Table 2). In the present study, from the perspective of health risk value, the average *HQ* of benzene (0.018) and ethylbenzene (6.7 × 10^−4^) was approximately one order of magnitude lower than that of other studies (Table 3) [8,21,64,65,67,69,71,72,73,75,76]. The HQ of toluene (0.003) was comparable to that in primary and secondary schools in Hanoi, Vietnam (0.001–0.003) [72] and residential homes in Louisiana, USA (0.003) [75]. In addition, the *LCR* values of benzene and ethylbenzene were one to three orders of magnitude lower than those reported in other studies (Table 3). These discrepancies may be due to the low concentration of benzene, toluene, and ethylbenzene measured in this study and the different parameters used in health risk assessments. For example, in this study, the human inhalation rate and body weight were set as 0.83 m^3^/h and 64.3 kg, whereas those adopted by Tran et al. [72] were approximately 0.65 m^3^/h and 80 kg, respectively. The non-carcinogenic risks of benzene, toluene, and ethylbenzene in many indoor living and learning places are generally less than one (Table 3), which is within the acceptable range. However, the carcinogenic risk value of benzene still exceeds the health risk threshold sometimes. For example, in the library of a university in New Delhi, India, the average *LCR* value of benzene was higher than 1 × 10^−6^ [21], and that in residential homes in Louisiana, USA was 1.4 × 10^−5^–4.9 × 10^−5^ [75]. Therefore, it is necessary to continue to pay attention to the health risks of benzene series in indoor environments in the future.

This study has some limitations. Although this study also evaluated the health risks of VOCs in canteens and libraries, which are also the main places for the daily activities of college students, due to the limited number of samples, the results may have large uncertainties. In addition, we did not consider other factors, such as humidity and the outdoor environment, which also have a significant impact on indoor VOCs [78,79,80]. Therefore, more samples need to be collected in these two places for more comprehensive analysis in the future.

## 5. Conclusions

In this study, a sampling campaign was conducted on campus at four different types of buildings (dormitory, canteen, library, and classroom) in different seasons, and a total of 116 VOCs were quantified using GC–MS/FID. The average indoor TVOC concentration was 203.3 ± 97.7 μg/m^3^, and the decreasing order of VOC concentrations at the four sampling points was as follows: dormitory (254.3 ± 100.8 μg/m^3^) > canteen (183.6 μg/m^3^) > classroom (154.0 ± 67.5 μg/m^3^) > library (139.4 μg/m^3^). Among the four sampling points, acetone (36.6–54.0 µg/m^3^) was the most abundant species, accounting for approximately 13.6−36.0% of the total VOCs. The dormitory, classroom, canteen, and library were free of substances that exceeded the non-carcinogenic risk limit. Acrolein showed the highest non-carcinogenic risk value at all four locations and was the most important non-carcinogenic risk substance. The carcinogenic risk of VOCs detected in the classroom, canteens, and library was acceptable. In the dormitory, the average *LCR* values of 1,2-dichloroethane were higher than 1.0 × 10^−6^ during the sampling period, indicating a possible cancer risk. Given the high health risks identified in the dormitory in this study, further research on associated sources and air quality improvement is recommended.

## Figures and Tables

**Table 1:** The non-cancer (*HQ*) and cancer (*LCR*) risks of the top 5 species in the four sampling sites.

Parameter	Species	Classroom	Species	Dormitory	Species	Canteen	Species	Library
*HQ*	Acrolein	0.07	Acrolein	0.11	Acrolein	0.10	Acrolein	0.05
	Trichloroethylene	0.02	Trichloroethylene	0.03	Trichloroethylene	0.03	Trichloroethylene	0.03
	Benzene	0.02	Benzene	0.02	Benzene	0.02	Benzene	0.02
	Chloroform	0.006	Chloroform	0.01	Chloroform	0.007	Toluene	0.004
	Toluene	0.002	1,2,4-Trimethylbenzene	0.005	Bromomethane	0.004	Chloroform	0.004
*LCR* (×10^−6^)	1,2-Dichloroethane	0.22	1,2-Dichloroethane	1.95	1,2-Dichloroethane	0.23	1,2-Dichloroethane	0.21
	Chloroform	0.08	Chloroform	0.18	1,2-Dibromoethane	0.20	1,2-Dichloropropane	0.06
	1,2-Dichloropropane	0.04	1,2-Dichloropropane	0.13	Chloroform	0.09	Chloroform	0.05
	1,2-Dibromoethane	0.04	1,2-Dibromoethane	0.06	1,2-Dichloropropane	0.08	Benzene	0.03
	Benzene	0.03	Bromodichloromethane	0.06	Benzene	0.03	Ethylbenzene	0.01

Note: The results from the canteen and library should be treated with caution due to only 1 sample being collected at each of these two places.

**Table 2:** Comparison of the concentration (μg/m^3^) of BTEX across different studies.

Environment Type	Region	Sample Place	Benzene	Toluene	Ethylbenzene	m,p-xylene	o-xylene	Reference
**University**	Hangzhou, China	Dormitory	1.66 ± 0.81	7.25 ± 4.09	1.71 ± 0.76	3.50 ± 1.86	1.45 ± 0.72	This study
		Classroom	1.40 ± 0.90	4.08 ± 3.13	1.16 ± 0.81	2.33 ± 1.87	1.01 ± 0.66	
	Wuhan, China	Classroom ^a^	97.5	11.4	13.7	3.5	3.0	[20]
	New Delhi, India	Library ^b^	7.2 ± 4.2	94.0 ± 70.1	10.1 ± 6.6	28.7 ± 31.7	13.1 ± 12.6	[21]
		Library ^c^	12.2 ± 7.9	66.7 ± 54.4	13.9 ± 8.6	22.2 ± 25.8	9.4 ± 11.8	
	Ankara, Turkey	Laboratories	15.5 ± 28.9	265 ± 591	5.63 ± 9.87	9.43 ± 13.3	1.6 ± 0.82	[9]
		Offices	6.42 ± 6.18	73.6 ± 57.4	5.31 ± 4.04	6.83 ± 3.98	1.72 ± 0.96	
		Classrooms	16.2 ± 16.5	44.1 ± 35.1	3.16 ± 2.86	5.25 ± 2.82	3.83 ± 6.64	
	Upper Silesia, Poland	Offices ^b^	1.13 ± 0.66	19.37 ± 26.63	2.32 ± 1.39	3.55 ± 2.35	0.87 ± 0.52	[66]
		Offices ^c^	0.46 ± 0.44	25.24 ± 27.77	3.96 ± 4.88	4.24 ± 4.25	1.43 ± 1.16	
	Bairrada, Portugal	Canteen	0.6–9.87	0.059–1.91	0.002–1.73	0.002–5.89	0.001–1.87	[56]
	Eskişehir, Turkey	Offices, demonstration room, conference hall	2.50 ± 1.0	149.93 ± 84.2	5.90 ± 4.1	10.13 ± 7.3	4.49 ± 2.6	[64]
	New Delhi, India	Commercial Shopping Complex	13.8 ± 8.9	67.1 ± 35.8	7.4 ± 4.1	40.6 ± 29.4	24.1 ± 21.1	[65]
**School**	Izmir, Turkey	Classroom	10.4	20.3	1.18	1		[67]
	Bari, Italy	Classroom	0.11–5.93	0.73–6.81	0.11–2.34	0.25–21.03		[68]
	Ho ChiMinh, Vietnam	Classroom	0.94 ± 1.6	7.7 ± 5.1	1.5 ± 0.77	3.1 ± 1.1		[69]
	Michigan, USA	Classroom	0.09	2.81	0.24	2.3		[70]
	Gran La Plata, Argentina	Classroom ^d^	2.26	12.05	1.44	5.24	1.71	[8]
		Classroom ^e^	2.51	8.70	1.63	7.13	2.45	
		Classroom ^f^	5.313	9.97	1.82	7.65	2.67	
	Gliwice, Poland	Classroom ^g^	1.37 ± 1.06	1.19 ± 0.95	2.11 ± 4.26	0.72 ± 0.66	3.31 ± 7.49	[71]
		Classroom ^h^	1.67 ± 1.25	1.63 ± 1.29	1.83 ± 3.23	0.87 ± 0.71	2.82 ± 5.99	
	Hanoi, Vietnam	Classroom	1.2–6.9	1.2–125	0.6–25.1	1.3–15.1	0.5–4.7	[72]
**Small enterprise**	Buenos Aires, Argentina	Chemical analysis laboratories	6.9	7.7				[73]
		Sewing workrooms		6.3				
		Electromechanical repair and car painting centers	59.2	243.1				
		Takeaway food shops		1.9				
		Photocopy center		3.3				
**Residential home**	Ashford, United Kingdom		0.5 (0.2–1.8)	1.5 (0.2–10.4)	0.8 (0.07–6.7)	1.5 (0.2–28.1)		[74]
	Louisiana, US		1.14 (0.04–13.57)	4.91 (0.84–66.22)	0.74 (0.29–8.65)	2.09 (0.63–35.36)		[75]
	Taiwan, China		7.0 ± 4.1	67.0 ± 36.7	17.1 ± 22.4	50.8 ± 66.1		[76]
**Hotel**	Michigan, USA	Guest room	0.9	2.4	0.3	0.2		[77]

Note: ^a^ the average level in the three universities; ^b^ in the cold season; ^c^ in the warm season; ^d^ schools in residential areas; ^e^ schools in urban areas; ^f^ schools in industrial areas; ^g^ classroom for older children; ^h^ classroom for younger children.

**Table 3:** Comparison of the non-cancer risk (*HQ*) and cancer risk (*LCR*) values of BTEX across different studies.

Environment Type	Region	Sample Place	Benzene		Toluene	Ethylbenzene		Reference
			*HQ*	*LCR*	*HQ*	*HQ*	*LCR*	
University	Hangzhou, China	Dormitory	0.019	1.16 × 10^−5^	0.004	7.89 × 10^−4^	3.85 × 10^−6^	This study
		Classroom	0.016	9.83 × 10^−6^	0.002 ± 0.002	5.33 × 10^−4^	2.6 × 10^−6^	
	New Delhi, India	Commercial Shopping Complex	0.0841 (0.0981)	1.97 × 10^−5^ (2.22 × 10^−5^)	0.0024 (0.0029)	0.0014 (0.0016)	1.50 × 10^−6^ (1.63 × 10^−6^)	[65]
	Eskişehir, Turkey	Stained Glass Workshop (for students)		2.06 × 10^−7^				[64]
	New Delhi, India	Library ^a^	0.248 (0.657)	5.27 × 10^−6^ (1.40 × 10^−5^)	0.0039 (0.0046)	0.0028 (0.0024)		[21]
		Library ^b^	0.42 (0.49)	8.93 × 10^−6^ (1.04 × 10^−5^)	0.0028 (0.0033)	0.0028 (0.0032)		
School	Izmir, Turkey	Classroom	0.31 ± 0.29	1.0 × 10^−6^ ± 1.2 × 10^−6^				[67]
	Ho Chi Minh, Vietnam	Classroom		0–4.53 × 10^−5^			0.08 × 10^−6^–0.32 × 10^−6^	[69]
	Gran La Plata, Argentina	Classroom ^c^		1.04 × 10^−6^				[8]
		Classroom ^d^		6.62 × 10^−7^				
		Classroom ^e^		1.10 × 10^−6^				
	Gliwice, Poland	Classroom		8.4 × 10^−6^–1.2 × 10^−5^				[71]
	Hanoi, Vietnam	Classroom	0.018–0.116	2.3 × 10^−5^–4.1 × 10^−5^	0.001–0.003	0.003–0.004		[72]
Residential home	Louisiana, US		0.145	1.4 × 10^−5^–4.9 × 10^−5^	0.003	0.002		[75]
	Taiwan, China			1.8 × 10^−4^				[76]
Small enterprise	La Plata city, Argentina	Chemical analysis laboratories		8.71 × 10^−5^	0.023			[73]
		Sewing workrooms		<2.43 × 10^−6^	0.004			
		Electromechanical repair and car painting centers		1.44 × 10^−4^	1.504			
		Takeaway food shops		<2.37 × 10^−6^	0.004			
		Photocopy center		<1.29 × 10^−6^	0.007			

Note: ^a^ in the cold season; ^b^ in the warm season; ^c^ schools in residential areas; ^d^ schools in urban areas; ^e^ schools in industrial areas. Values in parentheses are for females and values outside parentheses are for males.

## Data Availability

Some or all data that support the findings of this study are available from the corresponding author upon reasonable requests.

## Supplementary Materials

The following supporting information can be downloaded at: https://www.mdpi.com/article/10.3390/ijerph20105829/s1, Table S1: Sampling time and the number of samples collected in each place; Table S2: *RfD* of species used for calculating non-cancer risk and the Hazard Quotient (HQ) of each species in the dormitory, classroom, canteen, and library; Table S3: *SF* of species used for calculating lifetime cancer risk and the mean Lifetime Cancer Risk (*LCR*) of each species in the dormitory, classroom, canteen, and library; Table S4: The concentration (μg/m^3^) of each VOC detected in this study during the whole sampling period in the dormitory, classroom, canteen, and library; Figure S1: Relationship between temperature and the concentration level of TVOCs during the whole sampling campaign.
