# Supplementary material for: Indoor Volatile Organic Compounds: Concentration Characteristics and Health Risk Analysis on a University Campus
Source: Int J Environ Res Public Health. 2023 May 16;20(10):5829. doi: 10.3390/ijerph20105829 (PMC10218183; doi:10.3390/ijerph20105829)
Supplement: Supplementary file 1 [file ijerph-20-05829-s001.zip › ijerph-2272966-supplementary.pdf]

Supplementary Information

Indoor Volatile Organic Compounds: Concentration Characteristics and Health Risk Analysis on a University Campus

Shengjia Jin[1], Lu Zhong[1], Xueyi Zhang[1], Xinhe Li[1], Bowei Li[1,*], Xuekun Fang[1,2,*]

[1] College of Environmental & Resource Sciences, Zhejiang University, Hangzhou, Zhejiang 310058, China

[2] Center for Global Change Science, Massachusetts Institute of Technology, Cambridge, Massachusetts 02139, United States

* Corresponding author

Email:

fangxuekun@zju.edu.cn (Xuekun Fang)
libowei@zju.edu.cn (Bowei Li)

Table

Table S1. Sampling time and the number of samples collected in each place

Sampling time	Dormitory	Classroom	Library	Canteen
January (1.5-1.7)	3	3	1	1
April (4.27-4.29)	4	3	1	1
July (7.3-7.6&7.12-7.14)	6	6	0	0
October (10.25-10.28)	6	6	0	0

Table S2. RfD of species used for calculating non-cancer risk, and the Hazard Quotient (HQ) of each species at the dormitory, classroom, canteen, and library.

Substance	RfD	Dormitory		Classroom		Canteen*	Library*
	(mg/kg/d)	MEAN	SD	MEAN	SD	MEAN	MEAN
Naphthalene	0.02	2.14E-03	9.37E-04	1.19E-03	9.53E-04	1.20E-03	1.07E-03
Benzene	0.004	1.91E-02	9.34E-03	1.61E-02	1.03E-02	1.81E-02	1.68E-02
Toluene	0.08	4.17E-03	2.36E-03	2.35E-03	1.80E-03	3.46E-03	4.23E-03
Ethylbenzene	0.1	7.89E-04	3.49E-04	5.33E-04	3.72E-04	8.75E-04	7.58E-04
Styrene	0.2	1.53E-04	7.33E-05	5.38E-05	4.73E-05	7.80E-05	8.97E-05
1,3,5-Trimethylbenzene	0.04	1.32E-04	6.66E-05	9.00E-05	5.60E-05	2.04E-04	1.61E-04
1,2,4-Trimethylbenzene	0.01	4.50E-03	2.02E-03	2.10E-03	1.13E-03	2.92E-03	3.02E-03
1,2,3-Trimethylbenzene	0.01	7.93E-04	4.19E-04	4.33E-04	2.60E-04	4.71E-04	4.89E-04
Bromomethane	0.0014	2.52E-03	2.14E-03	1.81E-03	9.98E-04	4.00E-03	3.81E-03
Chloroform	0.01	1.48E-02	1.72E-02	6.40E-03	4.47E-03	7.30E-03	4.08E-03
1,1,1-Trichloroethane	2	9.78E-07	7.42E-07	7.38E-07	7.68E-07	1.86E-07	1.68E-07
Trichloroethylene	0.0005	3.30E-02	2.24E-02	1.77E-02	7.11E-03	2.71E-02	2.74E-02
1,1,2-Trichloroethane	0.004	2.21E-03	1.03E-03	1.27E-03	5.94E-04	2.73E-03	1.59E-03
Tetrachloroethylene	0.006	4.12E-03	2.75E-03	2.10E-03	1.10E-03	2.51E-03	2.30E-03
Dibromochloromethane	0.02	4.08E-04	5.42E-04	1.69E-05	9.94E-06	5.78E-05	3.03E-05
1,2-Dibromoethane	0.009	2.24E-04	1.57E-04	1.35E-04	1.70E-04	7.03E-04	2.73E-05
Chlorobenzene	0.02	2.06E-04	1.12E-04	2.10E-04	3.55E-04	4.13E-04	4.89E-04
Bromoform	0.02	1.38E-04	6.65E-05	7.82E-05	6.39E-05	1.59E-04	1.43E-04
1,2-Dichlorobenzene	0.09	2.42E-05	1.24E-05	1.70E-05	9.15E-06	8.99E-05	4.47E-05
tetrahydrofuran	0.9	4.47E-05	3.27E-05	3.61E-05	4.84E-05	1.95E-05	1.91E-05
Acrolein	0.0005	1.15E-01	4.82E-02	6.79E-02	3.68E-02	9.99E-02	4.87E-02
Acetone	0.9	2.76E-03	1.19E-03	1.87E-03	1.15E-03	1.32E-03	8.58E-04
Bromodichloromethane	0.02	1.50E-03	2.22E-03	8.15E-05	8.30E-05	1.11E-04	4.25E-05
2-Hexanone	0.005	1.97E-03	1.51E-03	1.53E-03	1.57E-03	6.20E-04	3.68E-04

Note:

* Due to only 2 samples being collected each in the canteen and the library, the results in both places have no SD.

Table S3. SF of species used for calculating lifetime cancer risk, the mean Lifetime Cancer Risk (LCR) and the maximum tolerable concentration (Tconc, mg/m^3) of each species at the dormitory, classroom, canteen, and library.

Substance	SF	Dormitory		Classroom		Canteen*		Library*	
		LCR	Tconc	LCR	Tconc	LCR	Tconc	LCR	Tconc
1,3-Butadiene	3.00E-05	6.45E-09	0.58	4.02E-09	1.36	6.16E-09	7.32	9.54E-09	17.15
Benzene	7.80E-06	3.18E-08	2.22	2.69E-08	5.21	3.02E-08	28.15	2.80E-08	65.97
Ethylbenzene	2.50E-06	1.05E-08	6.94	7.12E-09	16.26	1.17E-08	87.82	1.01E-08	205.84
Vinyl chloride	4.40E-06	1.40E-09	3.94	1.39E-09	9.24	2.57E-09	49.90	1.79E-09	116.95
1,1-Dichloroethane	1.60E-06	2.60E-09	10.85	3.26E-09	25.41	3.27E-09	137.23	2.73E-09	321.62
Chloroform	2.30E-05	1.82E-07	0.75	7.87E-08	1.77	8.97E-08	9.55	5.02E-08	22.37
1,2-Dichloroethane	2.60E-05	1.95E-06	0.67	2.16E-07	1.56	2.28E-07	8.44	2.11E-07	19.79
Trichloroethylene	4.10E-06	3.61E-09	4.23	1.94E-09	9.92	2.97E-09	53.55	3.00E-09	125.51
1,2-Dichloropropane	3.70E-05	1.30E-07	0.47	4.14E-08	1.10	8.25E-08	5.93	5.62E-08	13.91
1,1,2-Trichloroethane	1.60E-05	7.57E-09	1.08	4.33E-09	2.54	9.35E-09	13.72	5.44E-09	32.16
Tetrachloroethylene	2.60E-07	3.43E-10	66.74	1.75E-10	156.38	2.09E-10	844.47	1.92E-10	1979.22
1,2-Dibromoethane	6.00E-04	6.45E-08	0.03	3.88E-08	0.07	2.03E-07	0.37	7.87E-09	0.86
Bromoform	1.10E-06	1.63E-10	15.77	9.19E-11	36.96	1.87E-10	199.60	1.68E-10	467.82
1,4-Dichlorobenzene	1.10E-05	5.21E-08	1.58	2.62E-08	3.70	1.42E-08	19.96	6.28E-09	46.78
BenzylChloride	4.90E-05	4.46E-09	0.35	4.60E-09	0.83	1.02E-09	4.48	9.89E-10	10.50
Hexachloro-1,3-butadiene	2.20E-05	4.82E-09	0.79	4.31E-09	1.85	3.55E-09	9.98	2.10E-09	23.39
MTBE	2.60E-07	5.92E-10	66.74	3.86E-10	156.38	3.47E-10	844.47	2.82E-10	1979.22
1,4-dioxane	5.00E-06	9.18E-10	3.47	5.32E-10	8.13	3.63E-10	43.91	3.42E-10	102.92
Bromodichloromethane	3.70E-05	5.94E-08	0.47	3.22E-09	1.10	4.38E-09	5.93	1.68E-09	13.91

Note:

* Due to only 2 samples being collected each in the canteen and the library, the results at the canteen and library should be treated with caution.

Table S4 The concentration (μg/m^3) of each VOCs detected in this study during the whole sampling period at the dormitory, classroom, canteen, and library.

Group	Substance	Dormitory		Classroom		Canteen*	Library*
		Mean	SD	Mean	SD	Mean	Mean
Alkane	Ethane	4.23	1.99	3.33	1.83	4.91	5.50
	Propane	6.48	2.77	3.50	2.42	8.31	7.93
	iso-Butane	4.65	2.76	2.24	1.66	6.23	4.75
	n-Butane	5.48	2.23	2.10	1.32	3.82	3.40
	iso-Pentane	3.67	1.76	1.97	1.37	3.22	3.62
	n-Pentane	1.76	1.09	1.05	0.81	3.52	1.73
	n-Hexane	1.57	1.28	1.03	0.73	1.50	1.33
	n-Heptane	0.90	0.54	0.73	0.60	1.14	0.56
	Cyclohexane	0.53	0.41	0.43	0.38	0.36	0.37
	Cyclopentane	0.18	0.13	0.12	0.12	0.12	0.03
	2,3-Dimethylbutane	0.58	0.69	0.30	0.25	0.77	0.37
	2,2-Dimethylbutane	0.11	0.08	0.10	0.05	0.11	0.10
	2-Methylpentane	1.97	1.81	1.44	1.17	2.83	2.35
	3-Methylpentane	1.06	0.92	0.81	0.51	1.14	1.09
	2-Methylhexane	0.48	0.37	0.37	0.25	0.42	0.35
	3-Methylhexane	0.56	0.45	0.41	0.35	0.37	0.43
	2,3-Dimethylpentane	0.57	0.62	0.44	0.45	0.10	0.12
	2,4-Dimethylpentane	0.34	0.13	1.44	2.40	0.28	0.29
	2-Methylheptane	0.14	0.10	0.10	0.05	0.08	0.10
	2,3,4-Trimethylpentane	0.10	0.11	0.07	0.03	0.06	0.06
	2,2,4-Trimethylpentane	0.19	0.15	0.13	0.09	0.08	0.08
	3-Methylheptane	0.14	0.10	0.11	0.05	0.07	0.10
	Methylcyclohexane	0.34	0.21	0.27	0.11	0.28	0.32
	Methylcyclopentane	0.58	0.52	0.42	0.29	0.43	0.42
	n-Octane	0.59	0.40	0.28	0.18	0.62	0.27
	Nonane	0.31	0.11	0.25	0.16	0.23	0.20
	n-Decane	0.32	0.14	0.19	0.12	0.19	0.20
	n-Undecane	0.60	0.24	0.32	0.23	0.42	0.44
	n-Dodecane	1.59	0.71	0.45	0.19	0.52	0.57
Alkene	Ethylene	1.98	1.26	1.22	0.94	1.68	1.85
	Propylene	0.70	0.53	0.38	0.31	0.40	0.50
	1,3-Butadiene	0.09	0.05	0.05	0.04	0.08	0.13
	1-Butene	0.15	0.15	0.07	0.05	0.14	0.22
	cis-2-Butene	0.27	0.20	0.20	0.10	0.16	0.11
	trans-2-Butene	0.27	0.24	0.08	0.03	0.05	0.10
	1-Pentene	0.12	0.08	0.08	0.04	0.13	0.10

	cis-2-Pentene	0.15	0.11	0.09	0.08	0.02	0.02
	Isoprene	6.19	3.89	5.18	5.00	1.93	0.58
	trans-2-Pentene	0.07	0.09	0.03	0.02	0.02	0.02
	1-Hexene	0.32	0.37	0.12	0.09	0.06	0.10
Alkyne	Acetylene	1.10	0.78	1.03	0.93	1.27	1.31
Aromatic	Benzene	1.66	0.81	1.40	0.90	1.57	1.46
	Ethylbenzene	1.71	0.76	1.16	0.81	1.90	1.65
	Isopropylbenzene	0.12	0.05	0.11	0.04	0.12	0.11
	m/p-Xylene	3.50	1.86	2.33	1.87	3.57	3.05
	m-Diethylbenzene	0.25	0.20	0.05	0.03	0.06	0.03
	m-Ethyltoluene	0.29	0.17	0.21	0.15	0.37	0.31
	Naphthalene	0.93	0.41	0.52	0.41	0.52	0.47
	n-Propylbenzene	0.13	0.06	0.11	0.07	0.13	0.12
	o-Ethyltoluene	0.18	0.10	0.13	0.08	0.20	0.16
	o-Xylene	1.45	0.72	1.01	0.66	1.36	1.22
	p-Diethylbenzene	0.18	0.11	0.09	0.07	0.10	0.09
	p-Ethyltoluene	0.13	0.06	0.10	0.06	0.20	0.17
	Styrene	0.66	0.32	0.23	0.21	0.34	0.39
	Toluene	7.25	4.09	4.08	3.13	6.01	7.34
	1,2,3-Trimethylbenzene	0.17	0.09	0.09	0.06	0.10	0.11
	1,2,4-Trichlorobenzene	0.13	0.14	0.07	0.06	0.38	0.04
	1,2,4-Trimethylbenzene	0.98	0.44	0.46	0.24	0.63	0.66
	1,3,5-Trimethylbenzene	0.11	0.06	0.08	0.05	0.18	0.14
Halocarbon	1,1,1-Trichloroethane	0.04	0.03	0.03	0.03	0.01	0.01
	1,1,2,2-trachloroethane	0.02	0.01	0.02	0.01	0.03	0.00
	1,1,2-Trichloroethane	0.19	0.09	0.11	0.05	0.24	0.14
	1,1-Dichloroethane	0.66	0.28	0.83	0.21	0.83	0.69
	1,1-Dichloroethene	0.24	0.07	0.28	0.08	0.22	0.20
	1,2-Dibromoethane	0.04	0.03	0.03	0.03	0.14	0.01
	1,2-Dichlorobenzene	0.05	0.02	0.03	0.02	0.18	0.09
	1,2-Dichloroethane	30.53	27.56	3.38	1.70	3.57	3.30
	1,2-Dichloropropane	1.43	1.31	0.45	0.26	0.91	0.62
	1,3-Dichlorobenzene	0.44	1.00	0.16	0.50	0.21	0.02
	1,4-Dichlorobenzene	1.93	1.05	0.97	1.01	0.52	0.23
	BenzylChloride	0.04	0.03	0.04	0.05	0.01	0.01
	Bromoform	0.06	0.03	0.03	0.03	0.07	0.06
	Bromomethane	0.08	0.07	0.05	0.03	0.12	0.12
	CarbonTetrachloride	0.70	0.16	0.61	0.09	0.58	0.56
	Chlorobenzene	0.09	0.05	0.09	0.15	0.18	0.21

	Chloroethane	0.22	0.28	0.19	0.30	0.99	0.92
	Chloroform	3.22	3.73	1.39	0.97	1.59	0.89
	Chloromethane	2.18	0.86	2.18	1.33	2.13	2.07
	cis-1,2-Dichloroethene	0.04	0.02	0.04	0.02	0.04	0.02
	cis-1,3-Dichloropropene	0.02	0.01	0.02	0.02	0.01	0.00
	Dibromochloromethane	0.18	0.24	0.01	0.00	0.03	0.01
	Freon-11	1.41	0.27	1.52	0.17	1.73	1.81
	Freon-113	0.73	0.12	0.75	0.33	0.61	0.61
	Freon-114	0.14	0.09	0.11	0.09	0.15	0.19
	Freon-12	1.30	1.08	1.45	0.83	2.32	0.86
	Hexachloro-1,3-butadiene	0.09	0.05	0.08	0.06	0.07	0.04
	MethyleneChloride	9.07	4.85	7.54	4.84	11.20	10.73
	Tetrachloroethylene	0.54	0.36	0.27	0.14	0.33	0.30
	Trans-1,2-Dichloroethene	0.03	0.01	0.03	0.01	0.04	0.03
	trans-1,3-Dichloropropene	0.10	0.05	0.07	0.04	0.05	0.04
	Trichloroethylene	0.36	0.24	0.19	0.08	0.29	0.30
	Vinyl chloride	0.13	0.08	0.13	0.06	0.24	0.16
OVOC	1,4-dioxane	0.07	0.04	0.04	0.03	0.03	0.03
	2-Hexanone	0.21	0.16	0.17	0.17	0.07	0.04
	2-Pentanone	0.41	0.22	0.26	0.08	0.25	0.17
	2-propnol	10.72	20.61	9.88	31.96	5.19	7.10
	3-Pentanone	0.31	0.14	0.22	0.10	0.20	0.12
	4-methyl-2-pentanone	1.04	0.92	0.86	0.50	0.88	0.94
	Acetaldehyde	20.74	17.19	8.95	5.81	16.45	5.23
	Acetone	54.02	23.18	36.58	22.49	25.79	16.77
	Acrolein	1.25	0.52	0.74	0.40	1.08	0.53
	Bromodichloromethane	0.65	0.96	0.04	0.04	0.05	0.02
	Ethylacetate	13.79	11.10	5.72	4.87	7.74	8.52
	Hexanal	6.49	3.10	2.41	1.17	13.09	2.27
	Methacrolein	0.65	0.45	1.53	1.98	0.22	0.03
	Methyl Ethyl Ketone	3.44	1.23	3.04	1.19	3.32	5.42
	Methyl vinyl ketone	1.56	1.00	4.52	5.84	0.48	0.34
	Methyl methacrylate	0.08	0.04	0.06	0.08	0.04	0.08
	MTBE	0.93	0.69	0.60	0.46	0.54	0.44
	n-Butanal	2.09	0.66	2.08	1.30	1.68	1.55
	Pentanal	1.23	0.57	0.78	0.40	1.68	0.46
	Propanal	1.94	0.59	1.78	0.67	5.11	1.54

	Vinyl acetate	1.57	1.08	0.98	0.70	1.42	1.25
Other	Acetonitrile	1.60	1.52	1.10	0.67	1.73	0.58
	Carbondisulfide	1.14	0.69	2.71	6.15	0.68	0.77
	Tetrahydrofuran	0.87	0.64	0.71	0.95	0.38	0.37
TVOC		254.27	100.77	154.03	67.52	183.57	139.41

Note:

* Due to only 2 samples being collected each in the canteen and the library, the results in both places have no SD.

Figure

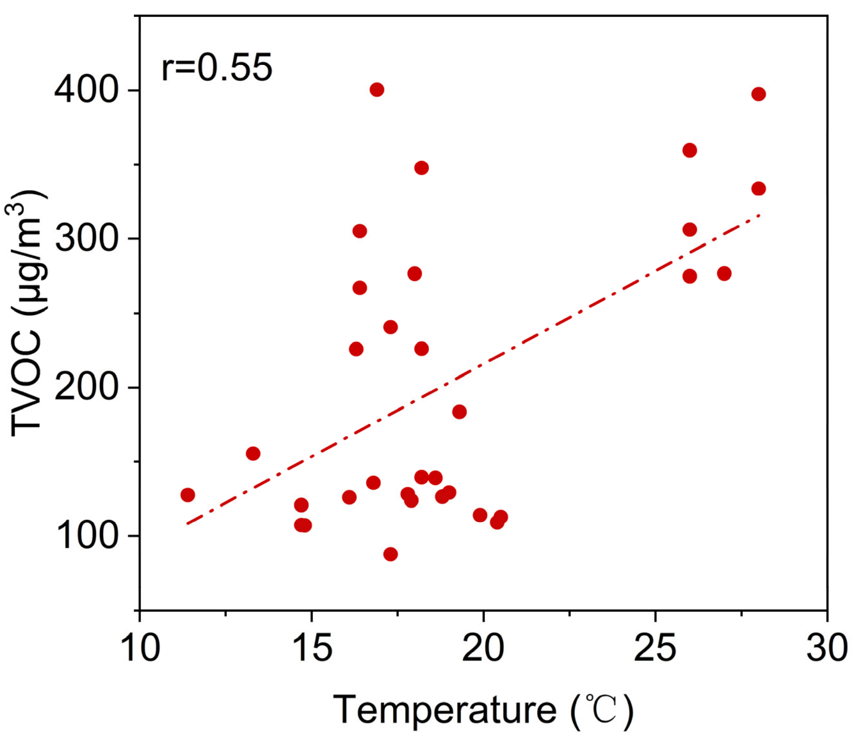

Figure S1 Relationship between temperature and the concentration level of TVOC during the whole sampling campaign.
